# Health-related quality of life of children and their parents 2 years after critical illness: pre-planned follow-up of the PEPaNIC international, randomized, controlled trial

**DOI:** 10.1186/s13054-020-03059-2

**Published:** 2020-06-16

**Authors:** José Hordijk, Sascha Verbruggen, Ilse Vanhorebeek, Fabian Güiza, Pieter Wouters, Greet Van den Berghe, Koen Joosten, Karolijn Dulfer

**Affiliations:** 1 Intensive Care Unit, Department of Pediatrics and Pediatric Surgery, Erasmus Medical Centre—Sophia Children’s Hospital, Dr. Molewaterplein 60, 3015 GJ Rotterdam, The Netherlands; 2 Clinical Division and Laboratory of Intensive Care Medicine, Department of Cellular and Molecular Medicine, KU Leuven, Herestraat 49, 3000 Leuven, Belgium

**Keywords:** Pediatric intensive care unit, Quality of life, Follow-up study, Parenteral nutrition, Parents

## Abstract

**Background:**

Pediatric intensive care unit (PICU) survivors are at risk for prolonged morbidities interfering with daily life. The current study examined parent-reported health-related quality of life (HRQoL) in former critically ill children and parents themselves and aimed to determine whether withholding parenteral nutrition (PN) in the first week of critical illness affected children’s and parents’ HRQoL 2 years later.

**Methods:**

Children who participated in the pediatric early versus late parenteral nutrition in critical illness (PEPaNIC) trial and who were testable 2 years later (*n* = 1158) were included. Their HRQoL outcomes were compared with 405 matched healthy controls. At PICU admission, children had been randomly assigned to early-PN or late-PN. In the early-PN group, PN was initiated within 24 h after PICU admission. In the late-PN group, PN was withheld for up to 1 week in the PICU. Parents completed the Infant Toddler Quality of Life Questionnaire (ITQOL; age 2–3 years) or the Child Health Questionnaire-Parent Form 50 (CHQ-PF50; age 4–18 years). Besides, they completed the Health Utility Index (HUI) and the Short Form Health Survey (SF-12) regarding their child’s and their own HRQoL, respectively.

**Results:**

For the total age group of 786 post-PICU survivors, parents reported lower scores for almost all HRQoL scales compared to healthy children. Age-specifically, younger critically ill children (2.5 to 3 years old) scored worse for growth and development and older children (4–18 years old) scored worse for role functioning and mental health. Parents’ own mental and physical HRQoL was comparable to that of healthy control parents. No HRQoL differences were found between children in the late-PN and those in the early-PN group.

**Conclusions:**

Parent-reported HRQoL of children 2 years after critical illness was impaired compared with healthy controls. In relation to their child’s HRQoL, parents reported impairments in emotions, personal time, and family activities; however, their own HRQoL was not impaired. Withholding PN in the first week during critical illness had no impact on longer-term HRQoL of the child.

**Trial registration:**

Clinical trials, NCT01536275. Registered 22 February 2012

## Background

Improvements in care for critically ill children have led to lower mortality rates in our pediatric intensive care units (PICUs) [1]. Nevertheless, a significant part of these surviving children will be confronted with increased morbidity after discharge from the hospital [1, 2]. Such morbidity carries a major burden on children and their families. Patient-reported outcomes (PROs) are an important source of information to assess these long-term consequences of critical illness on daily life [3]. PROs in the case of young children are reported by the parents and focus on the subjective evaluation of different domains regarding the perceived functioning of the child [4]. Health-related quality of life (HRQoL) is the most common PRO. It reflects the impact of health on the broad concept of quality of life, e.g., physical, mental, and social functioning, and provides insight in what the impairments mean for the daily life of the patient [5, 6].

We recently showed that parents reported lower HRQoL in PICU survivors, 6 months after PICU admission, compared with healthy children. Parents themselves reported better scores for physical HRQoL and worse scores for mental HRQoL compared with the general population [7]. This counterintuitive finding might reflect the short-term emotional impact on the parents/families of experiencing a life-threatening disease in their child [8]. However, little is known regarding longer-term HRQoL of critically ill children and their parents [9].

The pediatric early versus late parenteral nutrition in critical illness (PEPaNIC) multicenter, randomized controlled trial (RCT) showed that withholding supplemental parenteral nutrition (PN) during the first week in the PICU resulted in better short-term outcomes, with a reduced incidence of new infections, a shorter stay at the PICU, and reduced direct healthcare costs, compared with initiating parenteral nutrition on the day of admission to the PICU [10, 11]. Importantly, withholding PN for 1 week did not negatively affect survival, anthropometrics, health status, and neurocognitive development and even improved a few domains of parent-reported executive functioning, less externalizing behavioral problems, and improved visual-motor integration compared with children in the early-PN group, evaluated 2 years later [12]. In this secondary analysis, we first investigated parent-reported HRQoL of critically ill children as compared with healthy control children at 2-years follow-up and parents’ self-reported HRQoL as compared with parents of healthy control children. Secondly, we investigated whether the better long-term neurocognitive outcomes of children in the late-PN group are also reflected in a better HRQoL as compared with children who received early-PN in the PICU.

## Methods

### Design

This study is part of the pre-planned 2-year follow-up of the PEPaNIC trial that enrolled 1440 critically ill children admitted to the three participating PICUs (Belgium, the Netherlands, and Canada) between June 18, 2012, and July 27, 2015. The full study protocol with sample size calculation, short-term outcomes, and 2-year medical and neurocognitive outcomes have been published [10, 12, 13]. In summary, at PICU admission, children had been randomly assigned to early-PN or late-PN. In the early-PN group, PN was initiated within 24 h after PICU admission to supplement insufficient enteral caloric intake (whenever 80% of targeted calories per age and weight categories were not yet reached). In the late-PN group, PN was withheld for up to 1 week in the PICU, resulting in no PN in the majority of the children. After 1 week, for both groups equally, PN could be administered if necessary. When enteral nutrition covered ≥ 80% of calculated targets, supplemental PN was discontinued. Enteral nutrition was initiated early for both groups equally, and all patients received intravenous micronutrients until fully enterally fed.

### Participants at follow-up

Children who had participated in the PEPaNIC trial and who were alive and neurocognitively testable at 2-year follow-up were also eligible for the current study. Four hundred and five healthy control children, who had never been admitted to a neonatal or pediatric ICU, were recruited for a medical and neurocognitive assessment similar to that of the post-PICU patients. These children were demographically matched to the patients for age and gender. To control as much as possible for genetic and socio-economic/environmental background, siblings and relatives of the patients were preferably recruited into this control group, besides unrelated children recruited from the same geographic area.

### Procedure

From August 2014 through January 2018, all PICU survivors and their parents were first approached through a standardized patient information letter after screening for survival status was performed. When children’s neurocognitive functioning was not testable as determined by the physician and confirmed by the parents, they were not included in the analyses. Children who were neonates (0 to 6 months old) at the time of the PEPaNIC trial were tested at the age of 2.5 years due to the age-limits of the neurocognitive tests. When consent was obtained, they were subsequently contacted by phone to schedule an appointment for the follow-up assessment that was performed either at the hospital or at the patient’s home. Parents received the HRQoL measurements along with the confirmation letter of the appointment for follow-up assessment. One of the parents completed the questionnaires at home and handed them over to the researcher on the day of the follow-up assessment. Written informed consent was obtained from the parents or legal guardians and/or from the adolescent according to local regulations. The institutional review boards at each participating site approved this follow-up study.

### Health-related quality of life outcomes

The type of validated parent-reported questionnaires assessing the child’s HRQoL depended on the age of the child. Parents of patients 2.5–3 years old completed the Infant Toddler Quality of Life Questionnaire (ITQOL) about their child’s HRQoL [14], consisting of 103 items divided over 12 multi-item scales. Parents of patients 4–18 years old completed the Child Health Questionnaire-Parent Form 50 (CHQ-PF50) about their child’s HRQoL [15], consisting of 50 items divided into 11 multi-item scales and 4 single-item scales. The ITQOL and CHQ-PF50 are parallel forms of the same questionnaire and scores range from 0 (worst) to 100 (best) (see Additional file 1a and Additional file 5 for the psychometric characteristics of the questionnaires and a description of the subscales). Some subscales of these questionnaires are related to the impact of the health status of the child on the parents. Subsequently, all parents completed the Short Form Health Survey (SF-12) for assessment of their own HRQoL, independent of the health status of the child. The SF-12 consists of 12 items [16, 17] summarized in the “Physical Component Summary” (PCS) and “Mental Component Summary” (MCS) based on the US-derived summary scores with mean 50 and SD 10 and higher scores representing better HRQoL. Parents who had a child both in the patient group and in the control group completed the SF-12 twice.

The Health Utilities Index Mark 2 and 3 (HUI2 and HUI3) are based on the 15-item HUI questionnaire and are two different classification systems that together provide a combined view of the child’s HRQoL and provide a more objective way to measure the health status of the child. The HUI2 and HUI3 comprise respectively 6 or 7 function-attributes based on single items. Scores range from 1 (no functional limitations) to 4, 5, and 6 (severe functional limitations) and 1 weighted multi-attribute utility function with ranges of minus 0.36 (score worse than dead) to 1.00 (perfect health). In the current study, since children were matched with healthy control children, the HUI2 and HUI3 were assessed in children of all ages.

### Statistical analyses

The fraction of missing HRQoL data per variable was determined and analyzed to examine whether they were missing at random or not at random. In order to avoid selection bias, multiple imputation by chained equation (MICE) of HRQoL variables was performed when ≥ 70% of the data was available [18]. The number of imputed data sets was set equal to the percentage of missing data plus one. Predictors for missing values are described in Additional file 1b. The pooled estimates that take into account variation across imputations were reported.

To analyze the differences in HRQoL scales available for all ages between post-PICU patients and healthy control children and to investigate differences between patients randomly allocated to late-PN or early-PN during PICU stay, multivariable linear analyses were done on 21 imputed datasets with the beta-estimates reported as pooled results, preceded by a pooled univariable comparison with use of Student *t* test or Wilcoxon rank-sum test as appropriate. All multivariable analyses were adjusted for the baseline risk factors described in Additional file 1c and further for the short-term effects of the PEPaNIC trial as described in Additional file 1d. Sub-analyses were conducted for the HRQoL scales that were only available for a specific age range. Data are presented as beta-estimates with 95% confidence intervals (CI), means and standard deviations, or numbers and proportions, as appropriate. Statistical analyses were performed with the use of R version 3.4.3, MICE version 2.46.0, and JMP© version 13.0.0 (SAS Institute, Inc., Cary, NC). Two-sided *p* values ≤ 0.05 were considered statistically significant. To explore whether the results of the SF-12 were affected by the fact that some parents completed this questionnaire twice for two children participating in the study, one-way analyses of variance were done on three of the 21 imputed datasets as a sensitivity analysis. This trial is registered with ClinicalTrials.gov, NCT01536275.

## Results

**Fig. 1:**
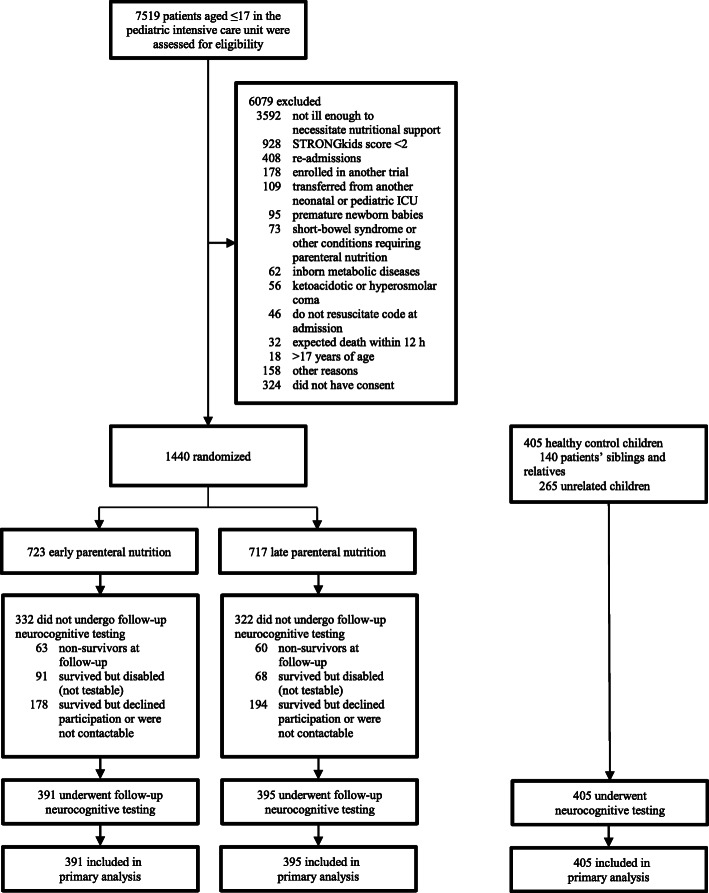
Flow diagram of study participants

Of the 1158 children who were alive and testable 2 years later, a total of 391 children in the early-PN group and 395 children in the late-PN group participated in the 2-year PEPaNIC follow-up study (Fig. 1; flow diagram of study participants). Demographic characteristics of the post-PICU children and matched healthy control children are shown in Table 1 (and Additional file 2). Children who were tested 2-years post-PICU admission were overall comparable to both the initial PICU children (Table 1) for demographics and patient characteristics upon PICU admission, as well as to the group of patients who survived, but declined participation or could not be reached (all *p* values > 0.15).

**Table 1:** Demographics of patients and healthy control children, post-randomization treatments in the PICU, and acute outcomes

	Tested populations		Total PICU population		Tested post-PICU population^**a**^	
	Healthy control children, ***N*** = 405	Post-PICU patients, ***N*** = 786	Early-PN, ***N*** = 723	Late-PN, ***N*** = 717	Early-PN, ***N*** = 391	Late-PN, ***N*** = 395
**Demographics**						
Age at 2-year follow-up (mean ± SEM), years	6.0 ± 0.2	5.7 ± 0.2	NA	NA	5.7 ± 0.2	5.6 ± 0.2
Male gender, no. (%)	219 (54.1)	455 (57.9)	415 (57.4)	412 (57.5)	230 (58.8)	225 (57.0)
Known non-Caucasian race, no. (%)^b^	33 (8.1)	63 (8.0)	50 (6.9)	33 (4.6)	38 (9.7)	25 (6.3)
Known non-European origin, no. (%)^b^	54 (13.3)	152 (19.3)	161 (22.3)	128 (17.9)	88 (22.5)	64 (16.2)
Known not exclusive Dutch or English language, no. (%)	76 (18.8)	184 (23.4)	122 (16.9)	106 (14.8)	95 (24.3)	89 (22.5)
Socio-economic status, no. (%)						
Educational level parents^c,d^						
Educational level 1	13 (3.2)	37 (4.7)	NA	NA	12 (3.1)	25 (6.3)
Educational level 1.5	23 (5.7)	54 (6.9)	NA	NA	28 (7.2)	26 (6.6)
Educational level 2	55 (13.6)	184 (23.4)	NA	NA	96 (24.6)	88 (22.3)
Educational level 2.5	76 (18.8)	131 (16.7)	NA	NA	60 (15.3)	71 (18.0)
Educational level 3	215 (53.1)	200 (25.4)	NA	NA	100 (25.6)	100 (25.3)
Educational level unknown	23 (5.7)	180 (22.9)	NA	NA	95 (24.3)	85 (21.5)
**Patient characteristics upon PICU admission**						
Infant (age < 1 year) at randomization, no. (%)	NA	363 (46.2)	328 (45.4)	325 (45.3)	177 (45.3)	186 (47.1)
STRONGkids risk level, no. (%)^e^						
Medium	NA	707 (89.9)	644 (89.1)	644 (89.8)	351 (89.8)	356 (90.1)
High	NA	79 (10.1)	79 (10.9)	73 (10.2)	40 (10.2)	39 (9.9)
PeLOD score, first 24 h in PICU (mean ± SEM)^f^	NA	20 ± 0.4	19.7 ± 0.4	20.1 ± 0.5	20 ± 0.6	20 ± 0.6
PIM3 score (mean ± SEM)^g^	NA	−3.5 ± 0.0	−3.2 ± 0.1	−3.2 ± 0.1	−3.4 ± 0.1	−3.5 ± 0.1
Diagnostic category, no. (%)^h^						
Surgical						
Cardiac	NA	339 (43.1)	279 (38.6)	268 (37.4)	173 (44.2)	166 (42.0)
Other	NA	249 (31.7)	211 (29.2)	215 (30.0)	125 (32.0)	124 (31.4)
Medical						
Respiratory	NA	83 (10.6)	99 (13.7)	96 (13.4)	39 (9.7)	45 (11.4)
Other	NA	115 (14.6)	134 (18.5)	138 (19.2)	55 (14.1)	60 (15.2)
Malignancy, no. (%)	0 (0.0)	42 (5.3)	51 (7.1)	33 (4.6)	26 (6.6)	16 (4.1)
Diabetes, no. (%)	0 (0.0)	1 (0.1)	3 (0.4)	0 (0.0)	1 (0.3)	0 (0.0)
Syndrome, no. (%)^i^	5 (1.2)	79 (10.1)	123 (17.0)	118 (16.5)	34 (8.7)	45 (11.4)
Known parental smoking between birth and PICU admission, no. (%)	NA	354 (45.0)	NA	NA	184 (47.1)	170 (43.0)
**Acute effects of randomization and post-randomization treatments in PICU**						
Duration of stay in the PICU (mean ± SEM), days	NA	7.4 ± 0.5	9.2 ± 0.8	6.5 ± 0.4	8.4 ± 0.9	6.4 ± 0.5
Patients who acquired a new infection in PICU, no. (%)	NA	105 (13.4)	134 (18.5)	77 (10.7)	66 (16.9)	39 (9.9)
Duration of mechanical ventilatory support (mean ± SEM), days	NA	4.7 ± 0.4	6.4 ± 0.7	4.4 ± 0.3	5.5 ± 0.7	3.9 ± 0.4
Number of days with hypoglycemia < 40 mg/dl (mean ± SEM), days	NA	0.1 ± 0.0	0.1 ± 0.0	0.2 ± 0.0	0.1 ± 0.0	0.2 ± 0.0
Duration of antibiotic treatment (mean ± SEM), days	NA	5.1 ± 0.5	6.7 ± 0.7	4.6 ± 0.3	5.8 ± 0.8	4.3 ± 0.5
Duration of hemodynamic support (mean ± SEM), days	NA	2.5 ± 0.3	3.0 ± 0.3	2.4 ± 0.2	2.6 ± 0.4	2.3 ± 0.3
Duration of treatment with opioids (mean ± SEM), days	NA	4.7 ± 0.3	6.1 ± 0.6	4.1 ± 0.2	5.4 ± 0.5	4.1 ± 0.3
Duration of treatment with benzodiazepines (mean ± SEM), days	NA	4.2 ± 0.3	5.4 ± 0.6	4.0 ± 0.3	4.5 ± 0.5	3.9 ± 0.5
Duration of treatment with hypnotics (mean ± SEM), days	NA	1.4 ± 0.2	1.8 ± 0.2	1.3 ± 0.1	1.6 ± 0.4	1.2 ± 0.1
Duration of treatment with alpha-2-agonists (mean ± SEM), days	NA	1.0 ± 0.2	1.1 ± 0.3	1.0 ± 0.2	0.9 ± 0.3	1.1 ± 0.3
Duration of treatment with corticosteroids (mean ± SEM), days	NA	1.2 ± 0.1	1.6 ± 0.2	1.3 ± 0.1	1.3 ± 0.2	1.0 ± 0.2

^a^No differences in demographics, allocation to late-PN or early-PN, and ICU/hospital primary/secondary study endpoints were observed between the tested post-PICU population (*N* = 786) and the group of patients who survived, but declined participation or could not be reached (*N* = 372) (all *p* > 0.15)

^b^Participants were classified according to race and geographical origin. These classifications were performed to capture ethnical and regional differences in the frequency of consanguinity

^c^The education level is the average of the paternal and maternal educational level and calculated based upon the 3-point scale subdivisions as made by the Algemene Directie Statistiek (Belgium; statbel.fgov.be/nl/) and the Centraal Bureau voor de Statistiek (the Netherlands; statline.cbs.nl): Low (= 1), middle (= 2), and high (= 3) educational level (Additional file 1f)

^d^For occupational level, see Additional file 2

^e^Scores on the Screening Tool for Risk on Nutritional Status and Growth (STRONGkids) range from 0 to 5, with a score of 0 indicating a low risk of malnutrition, a score of 1 to 3 indicating medium risk, and a score of 4 to 5 indicating high risk

^f^Pediatric Logistic Organ Dysfunction (PeLOD) scores range from 0 to 71, with higher scores indicating more severe illness

^g^Pediatric Index of Mortality 3 (PIM3) scores, with higher scores indicating a higher risk of mortality

^h^For more detailed information regarding the diagnostic categories, see Additional file 2

^i^A pre-randomization syndrome or illness a priori defined as affecting or possibly affecting neurocognitive development (Additional file 1e)

*Abbreviations*: *BMI* body mass index, *NA* not applicable (values only known when the patients were seen at follow-up, or not applicable for healthy control children), *PeLOD* pediatric logistic organ dysfunction score, *PICU* pediatric intensive care unit, *PIM3* pediatric index of mortality 3 score, *PN* parenteral nutrition, *SEM* standard error of the mean

Overall, critically ill children had worse outcomes at 2-year follow-up for parent-reported HRQoL compared with healthy control children (Table 2 and Additional File 3). For the total age group, differences between critically ill children and healthy control children were found in multivariable analyses for almost all parent-reported HRQoL multi-item scales, with differences of 5.4 to 27.2 points, with the exception of general behavior and family cohesion (Table 2). For the age-specific HRQoL scales, younger critically ill children (2.5 to 3 years old) scored worse for growth and development and older children (4–18 years old) scored worse for role functioning due to emotions/behavior and due to physical problems, and mental health compared with healthy control children. Critically ill children also scored worse on the parent-reported multi-attribute utility function on the dead-healthy scale for both HUI2 and HUI3 classifications, compared with healthy control children (Table 2 and Additional file 3). In multivariable analysis, lower scores were found on the HUI2 and HUI3 single utility scores for sensation, mobility, self-care, speech, and ambulation in patients compared with healthy controls (Table 2). Univariable analysis of HRQoL scores of parents of critically ill children reported for themselves a lower physical component score and mental component score compared with parents of healthy control children (Additional file 3). In the multivariable analysis, these differences were not statistically significant after adjusting for child and parent risk factors (Table 2). Sensitivity analyses without the data of *n* = 111 parents who completed the SF-12 two times or more showed no differences in results (Additional file 4).

**Table 2:** Multivariable linear and logistic regression analyses of the differences in HRQoL outcomes between study groups

HRQoL outcomes assessed at 2 years’ follow-up	No. (%) available data per outcome (***N*** = 1191)	Beta-estimate (95% CI) for the comparison of patients vs. controls, adjusted for risk factors^**a**^	***p*** value	Beta-estimate (95% CI) for the comparison late-PN vs. early-PN, adjusted for risk factors^**b**^	***p*** value
Parent-reported HRQoL in children (ITQOL & CHQ-PF50)					
Physical functioning	960 (81)	− 6.34 (− 8.59 to − 4.10)	**< 0.001**	1.32 (− 1.54 to 4.17)	0.36
Bodily pain	967 (81)	− 6.81 (− 9.54 to − 4.07)	**< 0.001**	1.20 (− 2.32 to 4.73)	0.50
General behavior	967 (81)	− 1.87 (− 3.88 to 0.14)	0.07	2.28 (− 0.01 to 4.57)	0.05
General health	961 (81)	− 27.20 (− 29.91 to − 24.49)	**< 0.001**	2.92 (− 0.47 to 6.31)	0.09
Change in health	965 (81)	12.94 (9.93–15.95)	**< 0.001**	− 0.88 (− 4.37 to 2.61)	0.62
Parental impact—emotional	964 (81)	− 7.92 (− 10.50 to − 5.33)	**< 0.001**	2.40 (− 0.80 to 5.60)	0.14
Parental impact—time	965 (81)	− 5.41 (− 8.07 to − 2.76)	**< 0.001**	3.05 (− 0.37 to 6.47)	0.08
Family activity	960 (81)	− 6.18 (− 8.78 to − 3.58)	**< 0.001**	3.58 (0.32–6.84)	**0.03**
Family cohesion	962 (81)	− 2.25 (− 4.97 to 0.47)	0.10	0.70 (− 2.41 to 3.81)	0.66
Parent-reported HRQoL in children 2.5 to 3 years only (ITQOL)					
Temperament and moods	548 (83)	− 1.14 (− 3.46 to 1.17)	0.33	− 0.33 (− 2.87 to 2.20)	0.80
Growth and development	547 (83)	− 3.09 (− 6.02 to − 0.15)	**0.04**	1.56 (− 1.52 to 4.65)	0.32
Getting along	543 (82)	− 0.25 (− 2.53 to 2.03)	0.83	1.56 (− 0.82 to 3.94)	0.20
Parent-reported HRQoL in children aged 4–18 years only (CHQ-PF50)					
Role functioning emotional/behavior	425 (80)	− 6.73 (− 12.86 to − 0.60)	**0.03**	− 1.26 (− 7.30 to 4.79)	0.68
Role functioning due to physical problems	424 (80)	− 7.87 (− 14.35 to − 1.39)	**0.02**	0.71 (− 5.00 to 6.43)	0.81
Mental health	425 (80)	− 5.28 (− 8.45 to − 2.11)	**0.001**	0.01 (− 3.62 to 3.65)	0.99
Self-esteem	426 (80)	− 2.34 (− 6.20 to 1.51)	0.23	1.08 (− 2.28 to 4.45)	0.53
Parent-reported HRQoL in children (HUI2; single utility scores)					
Sensation	959 (80)	− 0.03 (− 0.06 to 0.00)	**0.03**	0.02 (− 0.02 to 0.06)	0.30
Mobility	959 (80)	− 0.02 (− 0.04 to 0.01)	**0.007**	0.01 (− 0.02 to 0.023)	0.62
Emotions	962 (81)	− 0.01 (− 0.02 to 0.00)	0.06	0.00 (− 0.02 to 0.01)	0.43
Cognition	958 (80)	− 0.01 (− 0.02 to 0.00)	0.13	0.01 (− 0.01 to 0.03)	0.26
Self-care	960 (81)	− 0.04 (− 0.08 to 0.00)	**0.03**	0.01 (− 0.04 to 0.05)	0.83
Pain	961 (81)	0.00 (− 0.02 to 0.01)	0.56	0.00 (− 0.02 to 0.02)	0.66
Multi-attribute utility function on dead-healthy scale	944 (79)	− 0.04 (− 0.06 to − 0.02)	**< 0.001**	0.01 (− 0.01 to 0.03)	0.40
Parent-reported HRQoL in children (HUI3; single utility scores)					
Vision	958 (80)	0.00 (− 0.01 to 0.01)	0.96	0.00 (− 0.02 to 0.01)	0.77
Hearing	961 (81)	0.00 (− 0.02 to 0.02)	0.80	0.01 ( − 0.02 to 0.03)	0.57
Speech	960 (81)	− 0.04 (− 0.07 to 0.01)	**0.01**	0.02 (− 0.01 to 0.06)	0.21
Ambulation	959 (80)	− 0.04 (− 0.06 to − 0.01)	**0.002**	0.01 (− 0.02 to 0.04)	0.45
Dexterity	962 (81)	− 0.01 (− 0.04 to 0.01)	0.24	0.01 (− 0.03 to 0.04)	0.71
Emotions	962 (81)	− 0.007 (− 0.01 to 0.00)	0.05	0.00 (− 0.01 to 0.00)	0.41
Cognition	944 (79)	− 0.01 (− 0.03 to 0.00)	0.15	0.01 (− 0.02 to 0.04)	0.52
Pain	961 (81)	− 0.01 (− 0.03 to 0.01)	0.25	0.00 (− 0.02 to 0.03)	0.71
Multi-attribute utility function on dead-healthy scale	931 (78)	− 0.06 (− 0.09 to − 0.03)	**< 0.001**	0.02 (− 0.02 to 0.05)	0.33
HRQoL of parents (SF-12)					
Physical component score	938 (79)	− 0.38 (− 1.57 to 0.82)	0.53	0.66 (− 0.75 to 2.08)	0.36
Mental component score	938 (79)	− 0.88 (− 2.22 to 0.45)	0.19	1.12 (− 0.47 to 2.71)	0.17

Results are the combined beta estimates (95% confidence interval) from 21 datasets generated by multiple data imputation by chained equations under a “missing at random” assumption for the 786 post-PICU patients and 405 healthy control children. *p* values were considered statistically significant with two-tailed *p* values of less than .05 in which case they are expressed in bold. For a description of the subscales see, Additional file 5. Some parents did not complete all questions of a domain which resulted in differences between sample sizes on the subscales

^a^Estimates were adjusted for the following risk factors: age, center, race, gender, geographic origin, language, hand preference, history of malignancy, diabetes, a predefined “syndrome”, and the educational and occupational status of parents

^b^Estimates were adjusted for the following risk factors: age, center, race, gender, geographic origin, language, hand preference, history of malignancy, diabetes, a predefined “syndrome”, the educational and occupational status of parents, PIM3 score and PeLOD score upon PICU admission, STRONGkids risk category, and parental smoking behavior prior to PICU admission

*Abbreviations*: *HRQoL* Health-related quality of life, *PN* parenteral nutrition, *CI* confidence interval, *PICU* pediatric intensive care unit, *ITQOL* infant toddler quality of life questionnaire, *CHQ*-*PF50* Child Health Questionnaire-Parent Form 50, *HUI* healthy utility index, *SF*-*12* short form 12

Parents of critically ill children in the late-PN group overall reported comparable HRQoL scores as parents of critically ill children in the early-PN group, in univariable and multivariable analyses (Table 2 and Additional file 3).

## Discussion

### HRQoL of critically ill children versus healthy control children

Two years after children were included in the PEPaNIC trial, significant lower scores were found for post-PICU survivors compared with healthy control children on physical and general health-related HRQoL domains and single HUI scores. Besides, in younger post-PICU children, parent-reported growth and development was impaired. We previously showed that post-PICU children had worse outcomes for height, body weight, and neurocognitive development compared with healthy controls [12]. The present study showed that parents appear to assess these outcomes accurately in their child. Recent publications in short-term outcomes already reported these impairments in growth and development 6 months after critical illness [7, 19].

Impairments in physical HRQoL and in general health were still present in this study but parents reported more positive change in health on the single item scale “change in health” compared to 1year ago. In our previous 6-month PICU follow-up study [7], parents also reported lower physical HRQoL and also lower scores on psychosocial domains. At 2-year follow-up, only a few psychosocial domains in mainly older children remained impaired, whereas problems in other psychosocial multi-item domains, i.e., temperament and moods, getting along, and self-esteem, and single utility HUI scores emotions and cognition had normalized as compared with healthy control children. This is in line with a recent review that found that parent-reported HRQoL of children improved over time after critical illness [20]. Apparently, after PICU admission, the physical domain remained most impaired on the longer term. A review investigating studies into PICU survivors who experienced a cardiac arrest or acute respiratory distress syndrome also reported lower physical functioning and general health 5 and 10 years after critical illness [21]. This implicates that these problems remain during the development of the child and might interfere with daily life as they may lead to functional disabilities [9, 20]. It might be hypothesized that the timing of the follow-up after PICU admission is best within a couple of months to screen for growth and development impairments in younger children and to prevent mental health problems in older children [8].

### HRQoL of parents of critically ill children versus parents of healthy controls

In this study, parents reported that critical illness of their child had a negative impact on emotional wellbeing, personal time, and on activities with the family which is similar to a previous study in parents of children admitted to the PICU for cardiac arrest [22]. Interestingly, in the current study, when parents were asked about their own HRQoL, not directly in relation to the health of their child, they reported no differences compared with parents of healthy control children. Apparently, parents do experience some limitations in personal and family time and activities as a result of the health status of their child, but they do not experience this as an impairment of their own HRQoL. In contrast, in our previous study, 6 months after PICU admission, parents had higher scores on physical health and lower scores on mental health compared with the general population [7]. The higher psychosocial HRQoL in parents of critically ill children over time might be explained by a response shift. This occurs when parents’ appraisal of their own health status changes due to the adaptations they make to their child’s diminished health status, especially when their child has minor residual symptoms [9, 23].

### Late-PN versus early-PN during PICU admission

Withholding PN during the first week of critical illness had no impact on parent-reported HRQoL of the child compared with early administration of PN. This contrasts with the favorable outcomes on parent-reported executive functioning, in particular better inhibitory control, in critically ill children of the late-PN group [12]. This may be explained by the fact that HRQoL is a broader concept of the subjective evaluation of functioning on mental, physical, and social domains of daily life. Parents evaluate their child’s executive functioning as less developed compared to healthy peers but appear to retain the subjective feeling that their child is not impaired in overall daily functioning.

### Implications

Two years after PICU stay, children showed most impairments in physical HRQoL domains and therefore, follow-up programs should focus on these physical problems after PICU admission [20]. In addition, psychologists should screen on developmental problems in younger children and mental health problems in school-aged children. Those children who experience mental problems could be referred to a psychologist to prevent further problems in daily life. It is essential to ask parents to complete HRQoL outcomes after PICU admission to assess which specific needs exist with regard to daily functioning of the child and themselves. As the children included in this study are still relatively young, future research should be done on the longer-term to assess whether impairments remain on the longer term.

### Strengths and limitations

A strength of the current study is that the sample size was very large compared with other studies examining HRQoL in PICU survivors. Furthermore, we included the heterogeneous group of PICU patients and extensively adjusted the analyses for baseline and short-term follow-up risk factors for lower HRQoL. Hence, the results are generalizable to the impact of a PICU admission on long-term HRQoL [9], especially since the outcomes of PICU survivors were compared with those of healthy control children, matched for age and gender.

A limitation of the current study is the dependence on parent-reported outcomes since the majority of the children were too young to be able to assess their own HRQoL. Re-evaluation of the children when they are able to assess their own HRQoL in self-reports will provide further valuable information regarding HRQoL on the longer term after critical illness. Furthermore, children who were too disabled to test were not included in the current analyses. In our opinion, this would have introduced bias and limited the generalizability of the results since questionnaires assess to what extent children are able to participate in society. For example, parents are asked whether their child was limited by their health status in doing their homework or activities with friends in the last 4 weeks.

## Conclusions

Two years after critical illness, children showed an impaired parent-reported HRQoL, mainly on physical domains and general health. In relation to the critical illness of their child, parents reported impairments in emotions and personal time. However, parents’ own HRQoL appeared comparable to that of parents of healthy control children. Lastly, withholding PN in the first week during critical illness had no impact on HRQoL of the child on the longer term.

## Supplementary information

**Additional file 1.** Psychometric characteristics of the questionnaires and additional information about variables for analyses.

**Additional file 2.** Detailed demographic information regarding socioeconomic status and diagnostic category.

**Additional file 3.** Pooled univariable analyses of the differences in HRQoL outcomes between study groups.

**Additional file 4.** Sensitivity analyses of SF-12 outcomes of parents who have only one child in the study.

**Additional file 5.** Scales and score interpretation of the questionnaires.

## Data Availability

The datasets generated and/or analyzed during the current study are not publicly available due to additional papers that will be published on other related topics but are available from the corresponding author on reasonable request.

## Abbreviations

HRQoL: Health-related quality of life
PICU: Pediatric intensive care unit
PN: Parenteral nutrition
PEPaNIC RCT: Pediatric early versus late parenteral nutrition in critical illness randomized controlled trial
ITQOL: Infant Toddler Quality of Life Questionnaire
CHQ-PF50: Child Health Questionnaire-Parent Form 50
HUI: Health Utility Index
SF-12: Short Form Health Survey-12
PRO: Patient-reported outcomes
PCS: Physical component summary
MCS: Mental component summary
SD: Standard deviation
MICE: Multiple imputation by chained equation
CI: Confidence intervals
