# Supplementary material for: Health-related quality of life of children and their parents 2 years after critical illness: pre-planned follow-up of the PEPaNIC international, randomized, controlled trial
Source: Crit Care. 2020 Jun 16;24:347. doi: 10.1186/s13054-020-03059-2 (PMC7296688; doi:10.1186/s13054-020-03059-2)
Supplement: Supplementary file 1 — Additional file 1. Psychometric characteristics of the questionnaires and additional information about variables for analyses. [file 13054_2020_3059_MOESM1_ESM.docx]

**Additional file 1: psychometric characteristics of the questionnaires and additional information about variables for analyses**

**1a. Psychometric characteristics of the questionnaires**

The ITQOL has shown a good internal consistency (Cronbach’s alpha > 0.70) and moderate or adequate test–retest intra-class correlation coefficients (≥0.50; p < 0.001)(1). The CHQ-PF50 has shown good internal consistency, with Cronbach’s alpha for Dutch school children ranging from 0.39–0.96 for an average of 0.72 for the subscales(2). The SF-12 has shown good internal consistency, with Cronbach’s alpha coefficients of 0.72 to 0.89, and a test-retest reliability range between 0.73-0.86(3). The Health Utilities Index Mark 2 and 3 (HUI2 and HUI3) has been validated in pediatric populations and is considered to be reliable in children of 5–18 years of age(4).

**1b. List of variables used for multiple data imputation by chained equations**

Predictors for missing values were as following:
1) Demographics of patients and control children and patient characteristics upon PICU admission:

Centre, randomization to late-PN or early-PN, patient vs. controls, race, gender, geographic origin, language, hand preference, history of malignancy, history of diabetes, a predefined “syndrome” (Additonal file 1e), educational and occupational status of parents (Additional file 1f), diagnosis, PIM3 and PeLOD scores upon PICU admission, risk of malnutrition (STRONGkids category), parental smoking behavior prior to PICU admission, age at randomization, age group at randomization.

2) Acute effects of randomization and post-randomization treatments in PICU:

Acquisition of new PICU infections, duration of PICU stay, duration of mechanical ventilatory support,

hypoglycaemia, duration of treatment with haemodynamic support, antibiotics, corticosteroids, opioids, benzodiazepines, hypnotics and alpha-2-agonists.

3) Available 2-years neurocognitive and HRQoL variables:

Age, test location, height, weight, head circumference, composite endpoint “diagnosed with a somatic illness”, composite endpoint “diagnosed with a psychiatric illness”, composite endpoint “admitted to hospital for a medical or surgical reason”, clinical neurological examination, verbal IQ, performance IQ, total IQ, visual motor integration, reaction time left hand, reaction time right hand, within subject SD of reaction time left hand, within subject SD of reaction time left hand, number of unimanual taps right hand, number of unimanual taps left hand, number of valid alternating taps, number of valid synchronous taps, delta reaction time inhibition, delta number of errors inhibition, delta reaction time flexibility, delta number of errors flexibility, numbers memory span forward, numbers working memory backward, word pairs learning, word pairs immediate memory, word pairs delayed memory, word pairs recognition, pictures, dots learning, dots immediate memory, dots delayed memory, learning index, executive functioning as reported by parents/caregivers (inhibition, flexibility, emotional control, working memory, planning and organization, meta-cognition index, and total score), emotional and behavioral problems as reported by parents/caregivers (internalizing problems, externalizing problems, and total problems).

All HRQoL outcomes available for all ages, see Table 2. For HRQoL scales validated for a specific age-range (growth and development, temperament and moods, and getting along in children aged 3 years or younger, and role functioning emotional/behavioral, role functioning physical, mental health, and self-esteem in children who are 4 years or older), imputation was performed within these age-ranges only.

**1c. List of baseline risk factors for adjusting multivariable analyses**

All multivariable analyses were adjusted for the following risk factors: age, center, race, gender, geographic origin, language, hand preference, history of malignancy, diabetes, a predefined “syndrome” (Additional file 1e), and the educational and occupational status of parents (Additional file 1f). For the comparison between late-PN and early-PN groups, further adjustment was done for diagnosis and severity of illness Pediatric Index of Mortality (PIM3) and Pediatric Logistic Organ Dysfunction (PeLOD) upon PICU-admission, risk of malnutrition, and parental smoking behavior prior to PICU-admission.

**1d. List of short-term effects for adjusting multivariable analyses**

In order to investigate whether any eventual impact of late-PN versus early-PN on the long-term HRQoL outcomes might have been mediated by its acute effects on new PICU-infections and duration of PICU-stay, and thus possibly indirectly also number of post-randomization hypoglycemic events or the duration of post-randomization treatments such as mechanical ventilatory support, hemodynamic support, antibiotics, corticosteroids, opioids, benzodiazepines, hypnotics and alpha-2-agonists, explanatory statistical analyses were performed with further adjustment for these treatments.

**1e. Definition of “syndrome”**

A prerandomization syndrome or illness a priori defined as affecting or possibly affecting neurocognitive development, and which is subdivided in the following categories:

- Genetically confirmed syndrome or pathogenic chromosomal abnormality
- Clearly defined syndrome, association or malformation without (identified) genetic aberration
- Polymalformative syndrome of unknown etiology
- Clear auditory or visual impairment without specified syndrome
- Congenital hypothyroidism due to thyroid agenesis
- Brain tumor or tumor with intracranial metastatic disease
- Psychiatric disorder (e.g. autism spectrum disorder, (treatment for) attention deficit hyperactivity disorder)
- Severe medical disorder, not primarily neurologic, but suspected to alter psychomotor and/or mental performance
- Severe neonatal problem (e.g. severe asphyxia)
- Severe craniocerebral trauma or near-drowning
- Severe infectious encephalitis or drug-induced encephalopathy
- Infectious meningitis, encephalitis or Guillain-Barré
- Resuscitation and/or need for extracorporeal membrane oxygenation

**1f. Definition of educational and occupational level of parents**

**Educational level of parents**

The education level is the average of the paternal and maternal educational level, and calculated based upon the 3-point scale subdivisions as made by the Algemene Directie Statistiek (Belgium; statbel.fgov.be/nl/) and the Centraal Bureau voor de Statistiek (The Netherlands; statline.cbs.nl): Low (=1), middle (=2) and high (=3) educational level.

**Occupational level of parents**

The occupation level is the average of the paternal and maternal occupation level, which is calculated based upon the International Isco System 4-point scale for professions. In case one of the parents filled in two jobs in the questionnaire, the highest Isco code level was used. In case “unemployed”, “disabled”, “student”, or “housewife/houseman” was filled out, an Isco code level of 1 was given to that parent. When the parents described their profession as “employee”, “worker”, “liberal profession”, or “retired”, they were given an Isco code level of 2.
