# Supplementary material for: Health-related quality of life of children and their parents 2 years after critical illness: pre-planned follow-up of the PEPaNIC international, randomized, controlled trial
Source: Crit Care. 2020 Jun 16;24:347. doi: 10.1186/s13054-020-03059-2 (PMC7296688; doi:10.1186/s13054-020-03059-2)
Supplement: Supplementary file 2 — Additional file 2. Detailed demographic information regarding socioeconomic status and diagnostic category. [file 13054_2020_3059_MOESM2_ESM.docx]

**Additional file 2: Detailed demographic information regarding socioeconomic status and diagnostic category**

|  |  |  |  |  |  |  |  |
| --- | --- | --- | --- | --- | --- | --- | --- |
|  | **Tested populations** | |  | **Total PICU population** | | **Tested post-PICU population^a^** | |
|  |  |  |  |  |  |  |  |
|  | **Healthy control children**  **N=405** | **Post-PICU patients**  **N=786** |  | **Early-PN**  **N=723** | **Late-PN**  **N=717** | **Early-PN**  **N=391** | **Late-PN**  **N=395** |
|  |  |  |  |  |  |  |  |
|  |  |  |  |  |  |  |  |
| **Demographics** |  |  |  |  |  |  |  |
| Socioeconomic status - no. (%) |  |  |  |  |  |  |  |
| Occupational level parents^a^ |  |  |  |  |  |  |  |
| Occupational level 1 | 2 (0.5) | 10 (1.3) |  | NA | NA | 2 (0.5) | 8 (2.0) |
| Occupational level 1.5 | 25 (6.2) | 76 (9.7) |  | NA | NA | 33 (8.4) | 43 (10.9) |
| Occupational level 2 | 47 (11.6) | 127 (16.2) |  | NA | NA | 61 (15.6) | 66 (16.7) |
| Occupational level 2.5 | 26 (6.4) | 77 (9.8) |  | NA | NA | 44 (11.3) | 33 (8.4) |
| Occupational level 3 | 83 (20.5) | 121 (15.4) |  | NA | NA | 54 (13.8) | 67 (17.0) |
| Occupational level 3.5 | 40 (9.9) | 54 (6.9) |  | NA | NA | 32 (8.2) | 22 (5.6) |
| Occupational level 4 | 116 (28.6) | 108 (13.7) |  | NA | NA | 53 (13.6) | 55 (13.9) |
| Occupational level unknown | 66 (16.3) | 213 (27.1) |  | NA | NA | 112 (28.6) | 101 (25.6) |
| **Patient characteristics upon PICU admission** |  |  |  |  |  |  |  |
| Diagnostic category - no. (%) |  |  |  |  |  |  |  |
| Surgical |  |  |  |  |  |  |  |
| Abdominal | NA | 70 (8.9) |  | 53 (7.3) | 60 (8.4) | 34 (8.7) | 36 (9.1) |
| Burns | NA | 2 (0.3) |  | 5 (0.7) | 5 (0.7) | 1 (0.3) | 1 (0.3) |
| Cardiac | NA | 339 (43.1) |  | 279 (38.6) | 268 (37.4) | 173 (44.2) | 166 (42.0) |
| Neurosurgery-Traumatic brain injury | NA | 71 (9.0) |  | 63 (8.7) | 53 (7.4) | 39 (10.0) | 32 (8.1) |
| Thoracic | NA | 42 (5.3) |  | 34 (4.7) | 27 (3.8) | 23 (5.9) | 19 (4.8) |
| Transplantation | NA | 14 (1.8) |  | 7 (1.0) | 17 (2.4) | 4 (1.0) | 10 (2.5) |
| Orthopedic surgery-Trauma | NA | 23 (2.9) |  | 28 (3.9) | 26 (3.6) | 14 (3.6) | 9 (2.3) |
| Other | NA | 27 (3.4) |  | 21 (2.9) | 27 (3.8) | 10 (2.6) | 17 (4.3) |
| Medical |  |  |  |  |  |  |  |
| Cardiac | NA | 26 (3.3) |  | 30 (4.2) | 31 (4.3) | 10 (2.6) | 16 (4.1) |
| Gastrointestinal-Hepatic | NA | 3 (0.4) |  | 2 (0.3) | 4 (0.6) | 1 (0.3) | 2 (0.5) |
| Oncologic-Hematologic | NA | 8 (1.0) |  | 8 (1.1) | 7 (1.0) | 5 (1.3) | 3 (0.8) |
| Neurologic | NA | 44 (5.6) |  | 51 (7.1) | 52 (7.3) | 21 (5.4) | 23 (5.8) |
| Renal | NA | 0 (0.0) |  | 1 (0.1) | 1 (0.1) | 0 (0.0) | 0 (0.0) |
| Respiratory | NA | 83 (10.6) |  | 99 (13.7) | 96 (13.4) | 39 (9.7) | 45 (11.4) |
| Other | NA | 34 (4.3) |  | 42 (5.8) | 43 (6.0) | 18 (4.6) | 16 (4.1) |
|  |  |  |  |  |  |  |  |

a The occupation level is the average of the paternal and maternal occupation level, which is calculated based upon the International Isco System 4-point scale for professions (Additional file 1f)
