# Supplementary material for: Health-related quality of life of children and their parents 2 years after critical illness: pre-planned follow-up of the PEPaNIC international, randomized, controlled trial
Source: Crit Care. 2020 Jun 16;24:347. doi: 10.1186/s13054-020-03059-2 (PMC7296688; doi:10.1186/s13054-020-03059-2)
Supplement: Supplementary file 3 — Additional file 3. Pooled univariable analyses of the differences in HRQoL outcomes between study groups. [file 13054_2020_3059_MOESM3_ESM.docx]

**Additional file 3: Pooled univariable analyses of the differences in HRQoL outcomes between study groups**

|  |  |  |  |  |  |  |  |
| --- | --- | --- | --- | --- | --- | --- | --- |
|  |  | **Tested populations** | | | **Tested post-PICU population** | | |
|  |  |  |  |  |  |  |  |
| **HRQoL outcomes assessed at 2 years’ follow-up** | **No. (%) available data per outcome (N=1191)** | **Healthy control children**  **N=405** | **Post-PICU**  **patients**  **N=786** | **P-value** | **Early-PN**  **N=391** | **Late-PN**  **N=395** | **P-value** |
|  |  |  |  |  |  |  |  |
|  |  |  |  |  |  |  |  |
| Parent-reported HRQoL in children (ITQOL & CHQ-PF50) |  |  |  |  |  |  |  |
| Physical functioning | 960 (81%) | 100 (100-100) | 100 (88.9–100) | **<0.001** | 100 (86.7–100) | 100 (89.4-100) | 0.22 |
| Bodily pain | 967 (81%) | 91.7 (80-100) | 83.3 (66.7-100) | **<0.001** | 83.3 (66.7-100) | 83.3 (70-100) | 0.65 |
| General behavior | 967 (81%) | 76.7 (66.2-86.5) | 70 (56.5-83.3) | **<0.001** | 70 (55.8-81.2) | 71.9 (58.5-83.5) | 0.10 |
| General health | 961 (81%) | 86.3 (75.8-93.3) | 55 (36.3-69.6) | **<0.001** | 52.9 (35-68.3) | 55.8 (36.3-71.7) | 0.33 |
| Change in health | 965 (81%) | 50 (50-50) | 75 (50-100) | **<0.001** | 75 (50-100) | 75 (50-100) | 0.38 |
| Parental impact-emotional | 964 (81%) | 91.7 (82.1-100) | 82.1 (64.3-92.9) | **<0.001** | 82.1 (58.3-91.7) | 83.3 (66.7-92.9) | 0.11 |
| Parental impact-time | 965 (81%) | 100 (88.9-100) | 90.48 (76.2-100) | **<0.001** | 88.9 (69.1-100) | 90.5 (77.8-100) | **0.05** |
| Family activity | 960 (81%) | 91.7 (79.2-100) | 87.5 (66.7-100) | **<0.001** | 83.3 (66.7-100) | 87.5 (70.8-100) | 0.08 |
| Family cohesion ^a^ | 962 (81%) | 85 (60-85) | 85 (60-85) | **0.003** | 85 (60-85) | 85 (60-85) | 0.61 |
| Parent-reported HRQoL in children 2.5 to 3 years only (ITQOL) |  |  |  |  |  |  |  |
| Temperament and Moods | 548 (83%) | 79.9 (72.6-88.9) | 80.6 (70.8-90.3) | 0.50 | 80.6 (70.8-91.7) | 80 (70.8-88.9) | 0.48 |
| Growth and Development | 547 (83%) | 90 (77.5-95) | 77.5 (67.5-90) | **0.01** | 76.3 (60-89.4) | 77.5 (67.5-92.5) | 0.42 |
| Getting Along | 543 (82%) | 71.7 (66.2-80) | 70 (58.3-77.9) | 0.18 | 68.3 (58.3-76.7) | 70 (60-78.3) | 0.34 |
| Parent-reported HRQoL in children aged 4-18 years only (CHQ-PF50) | |  |  |  |  |  |  |
| Role functioning emotional/behavior | 425 (80%) | 100 (100-100) | 100 (89.9-100) | **<0.001** | 100 (88.9-100) | 100 (88.89-100) | 0.61 |
| Role functioning due to physical problems | 424 (80%) | 100 (100-100) | 100 (83.3-100) | **<0.001** | 100 (83.3-100) | 100 (83.33-100) | 0.68 |
| Mental Health | 425 (80%) | 80 (70-90) | 75 (60-85) | **<0.001** | 75 (60-85) | 75 (60-85) | 0.69 |
| Self-esteem | 426 (80%) | 79.2 (75-95.8) | 70.83 (58.3-83.3) | 0.06 | 70.83 (58.3-83.3) | 75 (62.5-87.5) | 0.40 |
| Parent-reported HRQoL in children (HUI2; single utility scores) |  |  |  |  |  |  |  |
| Sensation | 959 (80%) | 1 (0.87-1) | 0.87 (0.87-1) | **<0.001** | 0.87 (0.87-1) | 0.87 (0.87-1) | 0.51 |
| Mobility | 959 (80%) | 1 (1-1) | 1 (1-1) | **<0.001** | 1 (0.92-1) | 1 (1-1) | 0.07 |
| Emotions | 962 (81%) | 0.86 (0.86-1) | 0.86 (0.86-1) | **0.005** | 0.86 (0.86-1) | 0.86 (0.86-1) | 0.31 |
| Cognition | 958 (80%) | 1 (1-1) | 1 (1-1) | **<0.001** | 1 (1-1) | 1 (1-1) | 0.23 |
| Self-care | 960 (81%) | 1 (1-1) | 1 (0.85-1) | **<0.001** | 1 (0.85-1) | 1 (0.85-1) | 0.57 |
| Pain | 961 (81%) | 1 (1-1) | 1 (0.95-1) | **<0.001** | 1 (0.95-1) | 1 (0.95-1) | 0.43 |
| Multi-attribute utility function on Dead-healthy scale | 944 (79%) | 0.93 (0.88-1) | 0.87 (0.76-0.93) | **<0.001** | 0.85 (0.76-0.93) | 0.88 (0.78-0.93) | 0.39 |
| Parent-reported HRQoL in children (HUI3; single utility scores) |  |  |  |  |  |  |  |
| Vision | 958 (80%) | 1 (1-1) | 1 (1-1) | **0.02** | 1 (1-1) | 1 (1-1) | 0.57 |
| Hearing | 961 (81%) | 1 (1-1) | 1 (1-1) | **0.001** | 1 (1-1) | 1 (1-1) | 0.56 |
| Speech | 960 (81%) | 1 (0.67-1) | 0.82 (0.67-1) | **<0.001** | 1 (0.67-1) | 0.82 (0.67-1) | 0.63 |
| Ambulation | 959 (80%) | 1 (1-1) | 1 (1-1) | **<0.001** | 1 (0.83-1) | 1 (1-1) | **0.05** |
| Dexterity | 962 (81%) | 1 (1-1) | 1 (1-1) | **<0.001** | 1 (1-1) | 1 (1-1) | 0.18 |
| Emotions | 962 (81%) | 0.91 (0.91-1) | 0.91 (0.91-1) | **0.005** | 0.91 (0.91-1) | 0.91 (0.91-1) | 0.31 |
| Cognition | 944 (79%) | 1 (1-1) | 1 (0.86-1) | **<0.001** | 1 (0.86-1) | 1 (0.86-1) | 0.53 |
| Pain | 961 (81%) | 1 (1-1) | 1 (0.92-1) | **<0.001** | 1 (0.92-1) | 1 (0.92-1) | 0.40 |
| Multi-attribute utility function on Dead-healthy scale | 931 (78%) | 0.92 (0.79-1) | 0.79 (0.56-0.93) | **<0.001** | 0.79 (0.51-0.89) | 0.79 (0.57-0.93) | 0.36 |
| HRQoL of parents (SF-12) |  |  |  |  |  |  |  |
| Physical component score | 938 (79%) | 55.9 (52.6-57.8) | 54.4 (48.4-57.2) | **0.01** | 54.3 (47.9-57.1) | 54.5 (48.6-57.2) | 0.20 |
| Mental component score | 938 (79%) | 55.4 (48.3-60) | 53.5 (45.4-57.9) | **0.03** | 53.4 (44.5-57.8) | 53.49 (46-58.4) | 0.32 |
|  |  |  |  |  |  |  |  |

Results are the combined numbers (%) or medians (IQR) from 21 datasets generated by multiple data imputation by chained equations under a ‘missing at random’ assumption for the 786 post-PICU patients and 405 healthy control children. *P*-values were considered statistically significant with two-tailed *p*-values of less than .05 in which case they are expressed in bold. A higher score represents a better HRQoL. For ITQOL and CHQ-PF50: 0 is worst possible health state, 100 is best possible health. For the HUI2 and HUI3: scores range from 1 (no functional limitations) to 4, 5, and 6 (severe functional limitations) and 1 weighted multi-attribute utility function with ranges of minus 0.36 (score worse than dead) to 1.00 (perfect health). For SF-12: 50 is average with SD 10. For a description of the subscales see additional file 4. Some parents did not complete all questions of a domain which resulted in differences between sample sizes on the subscales.

a Medians are reported and are similar in the groups, means of the medians are as follows: healthy control children 85, post-PICU patients 77.3, Early-PN 77.9, Late-PN 76.7

Abbreviations: HRQoL, Health related quality of life; PN, parenteral nutrition; CI, confidence interval; PICU, pediatric intensive care unit; ITQOL, infant toddler quality of life questionnaire; CHQ-PF50, Child Health Questionnaire - Parent Form 50; HUI, healthy utility index; SF-12, short form 12
