# Supplementary material for: Health-related quality of life of children and their parents 2 years after critical illness: pre-planned follow-up of the PEPaNIC international, randomized, controlled trial
Source: Crit Care. 2020 Jun 16;24:347. doi: 10.1186/s13054-020-03059-2 (PMC7296688; doi:10.1186/s13054-020-03059-2)
Supplement: Supplementary file 4 — Additional file 4. Sensitivity analyses of SF-12 outcomes of parents who have only one child in the study. [file 13054_2020_3059_MOESM4_ESM.docx]

**Additional file 4: sensitivity analyses of SF-12 outcomes of parents who have only one child in the study**

N= 111 parents filled in the SF12 regarding themselves more than one time because they had 2 or more children participating in the follow-up study. Sensitivity analyses were done without the data of these parents (n=251 records) to explore differences between patients and healthy control children for parents’ own HRQoL. Three imputed datasets were randomly chosen from the 21 imputed datasets (dataset 1, 4, and 14). As shown below, differences between patients and controls remained statistically significant after excluding parents with 2 or more children in the follow-up study.

Dataset 1:

|  | | ^N^ | ^Mean^ | ^Std. Deviation^ | ^Std. Error^ | ^95% Confidence Interval for Mean^ | | ^Minimum^ | ^Maximum^ |
| --- | --- | --- | --- | --- | --- | --- | --- | --- | --- |
|  |  |  |  |  |  | ^Lower Bound^ | ^Upper Bound^ |  |  |
| ^SF12-Physical component score^ | ^Control^ | ^255^ | ^53,22^ | ^7,94^ | ^,50^ | ^52,24^ | ^54,20^ | ^19,48^ | ^65,73^ |
|  | ^Patient^ | ^704^ | ^49,91^ | ^10,42^ | ^,39^ | ^49,14^ | ^50,68^ | ^14,79^ | ^68,47^ |
| ^SF12-Mental component score^ | ^Control^ | ^255^ | ^53,03^ | ^9,77^ | ^,61^ | ^51,82^ | ^54,23^ | ^19,47^ | ^68,78^ |
|  | ^Patient^ | ^704^ | ^49,61^ | ^10,57^ | ^,40^ | ^48,83^ | ^50,39^ | ^13,15^ | ^73,48^ |

^Abbreviations: Std. Deviation, Standard Deviation; Std. Error, Standard Error; SF-12, Short Form 12
*^ *^p^*^-value= <.001^

Dataset 4:

|  | | ^N^ | ^Mean^ | ^Std. Deviation^ | ^Std. Error^ | ^95% Confidence Interval for Mean^ | | ^Minimum^ | ^Maximum^ |
| --- | --- | --- | --- | --- | --- | --- | --- | --- | --- |
|  |  |  |  |  |  | ^Lower Bound^ | ^Upper Bound^ |  |  |
| ^SF12-Mental component score^ | ^Control^ | ^255^ | ^52,63^ | ^9,44^ | ^,59^ | ^51,47^ | ^53,80^ | ^15,87^ | ^65,73^ |
|  | ^Patient^ | ^704^ | ^47,70^ | ^12,61^ | ^,48^ | ^46,77^ | ^48,64^ | ^14,79^ | ^68,47^ |
| ^SF12-Mental component score^ | ^Control^ | ^255^ | ^53,57^ | ^9,50^ | ^,59^ | ^52,40^ | ^54,74^ | ^19,47^ | ^68,78^ |
|  | ^Patient^ | ^704^ | ^51,31^ | ^9,98^ | ^,38^ | ^50,57^ | ^52,04^ | ^13,15^ | ^73,48^ |

^Abbreviations: Std. Deviation, Standard Deviation; Std. Error, Standard Error; SF-12, Short Form 12
*^ *^p^*^-value= <.001^

Dataset 14:

|  | | ^N^ | ^Mean^ | ^Std. Deviation^ | ^Std. Error^ | ^95% Confidence Interval for Mean^ | | ^Minimum^ | ^Maximum^ |
| --- | --- | --- | --- | --- | --- | --- | --- | --- | --- |
|  |  |  |  |  |  | ^Lower Bound^ | ^Upper Bound^ |  |  |
| ^SF12-Mental component score^ | ^Controle^ | ^255^ | ^52,91^ | ^8,45^ | ^,53^ | ^51,87^ | ^53,96^ | ^15,87^ | ^65,73^ |
|  | ^Patient^ | ^704^ | ^49,52^ | ^10,69^ | ^,40^ | ^48,73^ | ^50,31^ | ^14,79^ | ^68,47^ |
| ^SF12-Mental component score^ | ^Controle^ | ^255^ | ^52,14^ | ^10,47^ | ^,66^ | ^50,84^ | ^53,43^ | ^19,47^ | ^68,78^ |
|  | ^Patient^ | ^704^ | ^48,73^ | ^11,06^ | ^,42^ | ^47,91^ | ^49,55^ | ^13,15^ | ^73,48^ |

^Abbreviations: Std. Deviation, Standard Deviation; Std. Error, Standard Error; SF-12, Short Form 12
*^ *^p^*^-value= <.001^
