# Supplementary material for: Health-related quality of life of children and their parents 2 years after critical illness: pre-planned follow-up of the PEPaNIC international, randomized, controlled trial
Source: Crit Care. 2020 Jun 16;24:347. doi: 10.1186/s13054-020-03059-2 (PMC7296688; doi:10.1186/s13054-020-03059-2)
Supplement: Supplementary file 5 — Additional file 5. Scales and score interpretation of the questionnaires. [file 13054_2020_3059_MOESM5_ESM.docx]

**Additional file 5: scales and score interpretation of the questionnaires**

**5a. ITQOL scales and score interpretation***

| **Scale** | **Description low score** | **Description high score** |
| --- | --- | --- |
| Physical functioning (PF) | Child is considerably limited in performing physical activities such as eating, sleeping, grasping, and playing due to health problems | Child performs all types of physical activities such as eating, sleeping, grasping, and playing without limitations due to health problems |
| Growth and development (GD) | Parent is very dissatisfied with development (physical growth, motor, language, cognitive), habits (eating, feeding, sleeping) and overall temperament | Parent is very satisfied with development (physical growth, motor, language, cognitive), habits (eating, feeding, sleeping) and overall temperament |
| Bodily pain (BP) | Child has extremely severe, frequent and limiting bodily pain/discomfort | Child has no pain or limitations due to pain/discomfort |
| Temperament and moods (TM) | Child very often has certain moods and temperaments, such as sleeping/eating difficulties, crankiness, fussiness unresponsiveness and lack of playfulness and alertness | Child never has certain moods and temperaments, such as sleeping/eating difficulties, crankiness, fussiness unresponsiveness and lack of playfulness and alertness |
| General behavior (BE) | Parent believes child's behavior is poor and likely to get worse | Parent believes child's behavior is excellent and will continue as such |
| Getting along (GA) | Child very often exhibits behavioral problems, such as not following directions, hitting, biting others, throwing tantrums, and being easily distracted, while positive behavior, such as ability to cooperate, to appear sorry, and to adjust to new situations is seldom shown | Child never exhibits behavioral problems, such as not following directions, hitting, biting others, throwing tantrums, and being easily distracted, while positive behavior, such as ability to cooperate, to appear sorry, and to adjust to new situations is frequently shown |
| General health perceptions (GH) | Parent believes child's health is poor and likely to get worse | Parent believes child's health is excellent and will continue as such |
| Parental impact: emotional (PE) | Parent experiences a great deal of emotional worry/concern as a result of child's physical and/or psychosocial health and/or growth and development | Parent doesn't experience feelings of emotional worry/concern as a result of child's physical and/or psychosocial health and/or growth and development |
| Parental impact: time (PT) Family activities (FA) | Parent experiences a lot of limitations in time avail-able for personal needs due to child's physical and/or psychosocial health and/or growth and development | Parent doesn't experience limitations in time avail-able for personal needs due to child's physical and/or psychosocial health and/or growth and development |
| Family activities (FA) | The child's health and/or growth and development very often limits and interrupts family activities or is a source of family tension | The child's health and/or growth and development never limits and interrupts family activities or is a source of family tension |
| Family cohesion (FC) | Family's ability to get along is rated as ‘poor’ | Family's ability to get along is rated as ‘excellent’ |
| Change in health (CH) | Child's health is much worse now than 1 year ago | Child's health is much better now than 1 year ago |

* Cited by “The ITQOL/CHQ user's manual” (1,2)

**5b. CHQ-PF50 supplementary scales of the CHQ-PF50 and score interpretation**

| **Scale** | **Description low score** | **Description high score** |
| --- | --- | --- |
| Role functioning: Emotional / Behavior (REB) | Child is limited a lot in school work or activities with friends as a result of emotional or behavior problems | Child has no limitations in schoolwork or activities with friends as a result of emotional or behavior problems |
| Role functioning: Physical (RF) | Child is limited a lot in school work or activities with friends as a result of physical health | Child has no limitations in schoolwork or activities with friends as a result of physical health |
| Mental health (MH) | Child has feelings of anxiety and depression all of the time | Child feels peaceful, happy and calm all of the time |
| Self-esteem (SE) | Child is very dissatisfied with abilities, looks, family/peer relationships and life overall | Child is very satisfied with abilities, looks, family/peer relationships and life overall |

* Cited by “The ITQOL/CHQ user's manual”(1,2)
